# Characteristics of *Streptococcus agalactiae* causing urinary tract infections: Emergence of new sequence types ST74 and ST38 in Iran

**DOI:** 10.22038/IJBMS.2023.70337.15294

**Published:** 2023

**Authors:** Masome Razeghi, Chakameh Amini, Maryam Fazeli, Mehdi Goudarzi

**Affiliations:** 1 Infectious Diseases and Tropical Medicine Research Center, Shahid Beheshti University of Medical Sciences, Tehran, Iran; 2 Department of Biology, Science and Research Branch, Islamic Azad University, Tehran, Iran; 3 Department of Biology, North Tehran Branch, Islamic Azad University, Tehran, Iran; 4 ATMP Department, Breast Cancer Research Center, Motamed Cancer Institute, ACECR, Tehran, Iran; 5 Department of Microbiology, School of Medicine, Shahid Beheshti University of Medical Sciences, Tehran, Iran

**Keywords:** Drug resistance, Group B streptococci, Multilocus sequence typing, Multiplex polymerase chain – reaction, Polymerase chain reaction, Urinary tract infection, Vancomycin

## Abstract

**Objective(s):**

Difficult-to-treat *Streptococcus agalactiae* infections are increasingly described in patients with urinary tract infections (UTIs). This occurrence could be due to the production of virulence determinants. This study aimed to characterize the molecular features of *S. agalactiae* responsible for UTIs.

**Materials and Methods:**

In this cross-sectional study, 70 *S. agalactiae* isolated from UTIs were examined. Antibiotic susceptibility testing was performed using the disk diffusion method. All *S. agalactiae* isolates were confirmed by *atr* and *dltS* PCR assays. Virulence, alpha protein-like, and pilus island genes were detected by PCR. Isolates were characterized using the multilocus sequence typing method.

**Results:**

Multidrug resistance was observed in 80% of isolates. Five virulence profiles were detected, wherein *cylE*, *lmb*, *bca*, *rib* (35.7%), *cylE*, *lmb*, *alp3* (27.1%), and *cylE*, *lmb*, *bac*, *rib*, *alp2* (21.4%) were the most frequent detected profiles. *S. agalactiae* was isolated and categorized within three clonal complexes (CCs) including CC22 (40%), CC17 (25.7%), and CC23 (20%). The main sequence types (STs) found were ST22 (27.1%), ST23 (17.1%), ST17 (12.9%), ST31 (8.7%), ST40 (8.7%), ST74 (7.1%), ST48 (4.3%), ST890 (4.3%), ST189 (2.8%), ST38 (2.8%), ST52 (2.8%), and ST155 (1.4%). ST74 and ST38 were reported for the first time in Tehran-Iran.

**Conclusion:**

This study highlights the predominance of the CC22 lineage among *S. agalactiae* strains isolated from UTIs in Tehran, Iran, and highlights the significant penetration of this lineage into hospitals. MDR patterns among these strains appear to be becoming a major concern in the management of infections.

## Introduction

*Streptococcus agalactiae*, or Group B *Streptococcus* (GBS), is one of the most important nosocomial pathogens responsible for a variety of infections ranging from skin and soft tissue infections to urinary tract infections (UTIs), pneumonia, endocarditis, and meningitis in neonates and pregnant women. According to recently published data, GBS is associated with life-threatening infections and has a significant morbidity and mortality rate in affected cases (1). 

UTIs are among the most common infections, affecting about 30% of women during their lifetime. About 3% of these cases have more than one infection per year. It is well documented that Gram-negative bacteria, particularly *Escherichia coli*, are considered the most important causative agents of UTIs (2). Gram-positive bacteria are less prevalent compared to Gram-negative bacteria, which account for the majority of the global burden of UTIs. GBS is considered one of the causes of UTI and is responsible for 2–3% of cases ranging from asymptomatic bacteriuria, urethritis, cystitis, and pyelonephritis. Historically, it is well understood that the pathogenesis of GBS is related to a collection of virulence and antibiotic resistance factors expression (1, 2). In addition to the capsule, surface proteins, immune evasion factors, adhesion molecules, and toxins are also the major factors involved in the pathogenicity of GBS. Although the pathogenesis of this bacterium is associated with the expression of various virulence determinants, an important virulence factor identified in this microorganism is surface antigens belonging to alpha-like protein (Alp) family including alpha-C protein (*bca*), C beta protein (*bac*), Rib (*rib*), Alp2 (*alp2*), Alp3 (*alp3*), Alp4 (*alp4*), and epsilon (*epsilon/alp1*) (3-7). Examining the profile of these protein-coding genes increases the potential for subtyping GBS. There is evidence in the literature for sortase-dependent pilus-like structures and three pilus island (PI) alleles PI -1, PI -2a, and PI -2b on the surface of GBS that act as potential virulence factors (1, 8). Multilocus sequence typing (MLST) has been shown to have good potential for detecting and classifying GBS strains into different clonal complexes (CCs) and sequence types (STs) (5, 9). However, in recent decades, certain genotypes have been found to be associated with higher mortality rates, complications, and healthcare costs and may play an important role in the pathogenesis of GBS (1). Although several researchers have recently focused on understanding the molecular types and antibiotic resistance patterns of GBS in Iran, knowledge of the genetic diversity of this bacterium is still limited. To the best of our knowledge, this work investigates the genetic variability of GBS strain isolates from UTI and provides for the first time data on MLST of this bacterium in Tehran, the capital of Iran.

## Materials and Methods

***Sample collection and GBS isolation***

The project was conducted on 650 urine samples from patients undergoing testing for UTI. Over an 18-month period, from February 2021 to July 2022, a total of 70 GBS isolates were obtained and included in the study. This study was approved by the Ethics Committee of Shahid Beheshti University of Medical Sciences in Tehran, Iran (IR.SBMU.RETECH.REC.1400.252). According to the literature, the criterion for confirmation of UTI was a positive urine culture for GBS with a colony count of 105 CFU/ml or more. Bacteriological and biochemical methods such as Gramme stain, colony morphology, narrow zone beta-hemolytic colonies on 5% sheep blood, sodium hippurate hydrolysis, catalase, and CAMP test were used to confirm the isolates at the species level. Polymerase chain reaction (PCR) for the atr gene was performed for definitive identification (10). Finally, the confirmed GBS isolates were stored in Tryptic Soy Broth (TSB; Merck, Germany) containing 20% glycerol and 5% sheep blood at -70 °C for further analysis. 

***Determination of isolates susceptibility***

For evaluation of antibiotic susceptibility of *S. agalactiae* isolates towards the following antibiotics ampicillin, erythromycin, tetracycline, ceftriaxone, tetracycline, penicillin, vancomycin, chloramphenicol, clindamycin, cefotaxime, and levofloxacin (Mast Co., UK), Kirby Bauer disk diffusion assay was done following the Clinical and Laboratory Standards Institute (CLSI) criteria (11). The d-zone examination was performed for the detection of inducible resistance (ICR, iMLS_B_) GBS isolates with a double-disc test if resistance to erythromycin and susceptibility to clindamycin with a D-shaped zone around the clindamycin disk was formed. The existence of an inhibition zone with a circular shape (D test negative) is considered the MS phenotype (resistant to macrolide and susceptible to lincosamide). Resistance to both clindamycin and erythromycin was considered a constitutive resistance phenotype (CCR, cMLS_B_). Multidrug resistance (MDR) of GBS strains was specified as resistance to ≥ classes of antibacterial agents as earlier explained. *Streptococcus pneumoniae *ATCC 49619 was used as the control strain in each run test.

***Extraction of genomic DNA, detection of alpha-protein-like, and pilus island genes***

Using the aforementioned phenol-chloroform method, DNA was extracted from isolates that were GBS. For the assessment of DNA purity, the ratio of absorbance at 260 and 280 nm was measured using a spectrophotometer. Screening of an array of alpha-protein-like and pilus island genes including *bca *(alpha C protein)*,* *alp2/3 *(C alpha-like protein 2/3),* rib* (cell surface rib protein)*, eps* (epsilon protein gene)*,* and *alp4 *encoding genes was performed using multiplex PCR assay with five pairs of primers as previously described by Creti *et al* (3). To identify each of the *lmb *(human laminin-binding protein),* cylE *(beta-hemolysin), and *bac* genes (cell surface beta C protein), a PCR assay was applied as earlier described (5). The PI-1, PI-2a, and PI-2b mediated virulence was detected by using PCR as described elsewhere (12). 

***Screening of macrolide and tetracycline resistance determinants***

The detection of the *erm *(A), *erm *(B), and *mef *(A/E), as macrolide-mediated resistance genes and *tet *(M), *tet *(O), *tet *(K), *tet *(L), and *tet *(S) as tetracycline resistance genes was performed using PCR protocol described by Palmeiro *et al* (13, 14).

***Multilocus sequence typing (MLST)***

MLST technique was carried out according to the protocol described in the MLST database website (http://pubmlst.org/sagalactiae/). Sequence types (STs) were assigned by MLST assay based on sequencing of internal fragments (*adhP*,* pheS*,* atr*,* glnA*,* sdhA*, *glcK*, and* tkt*) and submission of their sequences to the online MLST database website and goeBURST algorithm. The previously undiscovered allele and STs were deposited in the S. *agalactiae* MLST database as a new type. The STs were classified into different clonal complexes (CCs) based on similar allele profiles.

## Results

***Isolation and antimicrobial resistance profiles***

In the current survey, 70 GBS strains were isolated from urine samples. Out of which, 61 (87.1%) were from male participants and 9 (12.9%) were from female participants with a mean age of 39 y (range, 18–65 years). The majority of isolates were obtained from patients aged 30–45 (64.3%) years. According to the Kirby Bauer assay, all the isolates were susceptible to ampicillin, penicillin, vancomycin, and ceftriaxone. Overall, resistance to all antibiotics was not detected among tested isolates. The resistance rate of erythromycin was the highest (88.6%; 62/70) followed by tetracycline (80%; 56/70), clindamycin (40%; 28/70), chloramphenicol (35.7%; 25/70), levofloxacin (21.4%; 15/70), and cefotaxime (7.1%; 5/70). In total, 80% (56/70) of isolates were found MDR. As given in Table 1 eight resistance profiles were detected, wherein ERY, CLI, TET (40%; 28/70), ERY, LEV, TET (14.3%; 10/70), and ERY, TET, CHO (14.3%; 10/70) were the three most frequently detected profiles. In the present study 24 (34.3%), 28 (40%), and 10 (14.3%) of the isolates were confirmed as ICR, CCR, and M phenotypes.

***Virulence, resistance, and PI encoding genes detection***

Regarding tetracycline resistance determinants, *tet*(M) was the most prevalent gene representing 70% (49/70) of isolates followed by *tet*(O) (14.3%; 10/70), *tet*(K) (7.1%; 5/70), *tet*(L) (4.3%; 3/70), and *tet*(S) (2.9%; 2/70). Among the 56 tetracycline-resistant isolates, 6 isolates (10.7%) carried the *tet*(M) and *tet*(O) genes, 3 isolates *tet*(M) and *tet*(L) (5.4%), 2 isolates *tet*(M), *tet*(K) and *tet*(O) (3.6%), and 2 isolates *tet*(S) and *tet*(O) (3.6%) simultaneously. At least one *tet* gene was identified in each tetracycline resistance isolate. Macrolide resistance was predominantly due to *erm*(B), which was detected in 44 isolates (62.9%) of the isolates followed by *erm*(A) in 24 isolates (34.3%) and *mef *(A/E) in 13 isolates (18.6%). Our findings indicated that of these strains, 25.8% (16/62) carried both *erm*(A) and *erm*(B), and 4.8% (3/62) carried both *erm*(B) and *mef *(A/E). Intriguingly, ICR isolates carried *erm*(A) (100%) and *erm*(B) (66.7%) while CCR isolates carried *erm*(B) (100%, 28/28) and *mef*(A/E) (10.7%, 3/28). Specifically, all isolates with M phenotype carried only *mef* (A/E).

The multiplex PCR assay of surface protein antigen genes revealed that out of 70 isolates, 45 isolates (64.3%) had *rib* gene, 36 isolates (51.4%) *bca*, 26 isolates (37.1%) *alp2*, 25 isolates (35.7%) *alp3*, 20 isolates (28.6%) *bac*, and 6 isolates (8.6%) *alp4*. All isolates were found to carry *cylE* and *lmb* genes. Regarding virulence encoding genes, five profiles were detected, wherein *cylE*, *lmb*, *bca*, *rib* (35.7%; 25/70), *cylE*, *lmb*, *alp3* (27.1%; 19/70), and *cylE*, *lmb*, *bac*, *rib*,* alp2* (21.4%; 15/70) were the three most frequently detected profiles. Regarding pilus island genes, the highest PI rate was recorded for PI-2a (75.7%; 53/70), followed by PI-1 (70%; 49/70) and PI-2b (24.3%; 17/70). Interestingly, 39 isolates (55.7%) carried PI-1 and PI-2a, and 10 isolates (14.3%) carried PI-1 and PI-2b simultaneously, while 14 isolates (20%) and 7 isolates (10%) were found to carry PI-2a and PI-2b lonely, respectively. The findings indicated that the 21 isolates carried at least one of the 3 PI types.

***MLST***

**Figure 1:**
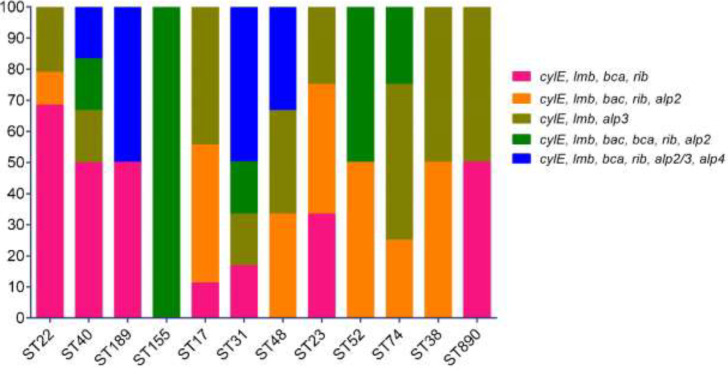
Distribution of alpha-protein-like genes in different STs of GBS strains obtained from urine samples of patients with UTIs

**Figure 2:**
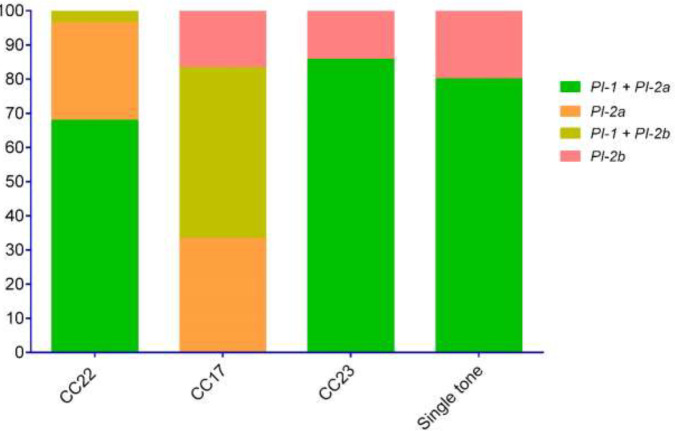
Distribution of pilus island genes in different CCs of GBS strains of obtained from urine samples of patients with UTIs

According to the MLST method, isolates were assigned to particular STs among tested isolates, including ST22 (27.1%; 19/70), ST23 (17.1%; 12/70), ST17 (12.9%; 9/70), ST31 (8.7%; 6/70), ST40 (8.7%; 6/70), ST74 (7.1%; 5/70), ST48 (4.3%; 3/70), ST890 (4.3%; 3/70), ST189 (2.8%; 2/70), ST38 (2.8%; 2/70), ST52 (2.8%; 2/70), and ST155 (1.4%; 1/70), which were categorized into 3 CCs including CC22 (40%; 28/70), CC17 (25.7%; 18/70), and CC23 (20%; 14/70) and 3 single tones (14.3%; 10/70). As shown in Table 1 ICR phenotypes were assigned to seven particular types as follows: ST22 (14.3%; 10/70), ST40 (8.6%; 6/70), ST189 (2.9%; 2/70), ST74 (5.7%; 4/70), ST38 (2.9%; 2/70), ST890 (5.7%; 4/70). M phenotypes were detected in ST22 (12.9%; 9/70) and ST155 (1.4%; 1/70). Near half of the isolates with CCR phenotype belonged to ST23 (17.1%; 12/70) while the rest of isolates belonged to ST17 (12.9%; 9/70), ST31 (5.7%; 4/70), and ST48 (4.3%; 3/70). As previously mentioned, five virulence profiles were identified in the 70 collected GBS isolates, the predominant profiles among CC22 isolates were *cylE*, *lmb*, *bca*, and *rib,* while in CC17 isolates *cylE*, *lmb*, *alp3*, and in CC14 isolates *cylE, lmb, bac, rib, alp2 *were the most prevalent detected virulence profile. The distribution of alpha-protein-like and pilus island genes in different STs obtained from urine samples is presented in Figure 1. Overall, PI types were strongly correlated with specific ST. All ST22 (19 isolates), ST23 (12 isolates), ST74 (4 isolates), and ST890 (3 isolates) were PI-1 + PI-2a, all ST40 (6 isolates) and ST189 (2 isolates) were PI-2a, all ST155 (1 isolate) and ST17 (9 isolates) were PI-1 + PI-2b and ST52 (2 isolates) and ST38 (2 isolates) were PI-2b. The distribution of pilus island genes in different CCs of GBS strains is presented in Figure 2.

## Discussion

Our analysis showed that all GBS strains lacked resistance to ampicillin, penicillin, vancomycin, and ceftriaxone. This finding is consistent with reports by other investigators (15-17) confirming that penicillin therapy is strongly recommended as the treatment of choice for GBS infections. However, several studies in Japan (18), Iran (19), and America (20) reported different percentages of decreased penicillin susceptibility. In the present work, data showed that the majority of GBS isolates (80%) were resistant to tetracycline. Our results are consistent with reports from Italy (80%) (6) and Brazil (83%) (21), but lower than previous studies in Iran (95%) (17) and Ethiopia (93.8%) (22). The high prevalence of tetracycline-resistant GBS isolates may be due to the unrestricted and unscheduled administration of this antibiotic, the lack of suitable alternatives to tetracycline, different attitudes toward antimicrobial protocols, and the proliferation of tetracycline-resistant specific clones.

Moreover, according to the present results, *tet*(M) was the predominant gene in tetracycline-resistant isolates, which is consistent with studies on 146 clinical isolates of GBS in Iran, where tet(M) was found to be the most common resistance gene (92.7%) (4, 15). Our observations on other tetracycline resistance genes, including tet(O), *tet*(K), tet(L), and *tet*(S), are consistent with other studies that confirmed the low prevalence of these genes compared with tet(M), which may be due to low expression or transmissibility of these genes (23), but further studies are needed to confirm this conclusion.

In the present study, the prevalence of erythromycin resistance in GBS isolates was reported to be 88.6%. The rate reported in this study was higher than previous studies from Iran (28.1%) (15), China (69%) (5), Italy (19.5%) (6), Brazil (11%) (21), Spain (23.4%) (24), and Taiwan (68.1%) (25). In this study, we found that 40% of the isolates were resistant to clindamycin, which was lower than the resistance rates reported in Taiwan (65.9%) (25), China (50.6%) (5), and Iran (47%) (17), and higher than the rates reported in Brazil (5%) (21), Japan (12.6%) (26), and Spain (20.6%) (24). Overall, the reason for this high erythromycin resistance rate is not well understood, but it appears to be related to inadequate management of antibiotic administration programs, improper unrestricted policies, and widespread use of this antibiotic.

The prevalence of clindamycin in our study was 40%. Previous studies from China (50.6%) (5), Brazil (5%) (21), Spain (20.6%) (24), and Taiwan (65.9%) (25) reported varying rates of resistance to clindamycin in GBS strains. However, the increasing resistance to clindamycin can be attributed to the use of this antibiotic for prophylaxis and treatment of patients with penicillin allergy, regional differences in dosing practices, public health policies, and the spread of specific clindamycin-resistant clones. All GBS isolates with CCR carried erm(B) and 3 isolates carried erm(B) + *mef*(A/E) simultaneously. Our observations are consistent with a study (pdf7) in Italy, where a high prevalence of the CCR phenotype was found, and the erm(B) gene was the major resistance mechanism. In a recent study, the M phenotype encoded by *mef*(A/E) was detected in GBS isolates with a prevalence of 14.3%. Similarly, in Taiwan, a study showed an M phenotype rate of 49.2% in GBS strains that were all carriers of *mef*(A) (27). The high frequency of the *mef*(A/E) gene (14.3%) in the present study contrasts with reports from Iran (2.4%) (15) and Tunisia (2.2%) (28). The GBS strains with ICR phenotype identified in the present study were mainly *erm*(A) and *erm*(B) positive. The co-occurrence of *erm*(B) and *erm*(A) in GBS isolates with ICR phenotype has been described in several countries such as Brazil (21), Taiwan (27), and China (23).

It has been documented that surface proteins of GBS are adhesins that play an important role in immune escape. In the present study, six major virulence profiles encoding surface proteins involved in invasion, immune evasion, or adhesion were identified, with *cylE*, *lmb*, *bca*, and *rib* (35.7%) being the most abundant, which is quite similar to a report from China (29) and one from Iran (17). It is worth noting that all isolates carried the virulence factors cylE and *lmb*. This hemolytic factor is an important factor in the colonization and pathogenesis of GBS. Similar results were reported regionally from Iran (19) and China (23). Results of alpha-protein-like genes detection also indicated a high prevalence rate of the *rib* gene (64.3%) followed by *bca *(51.4%), *alp2 *(37.1%), *alp3 *(35.7%), *bac *(28.6%), and *alp4* (8.6%), which was consistent with the earlier study performed by Sadeh *et al.* in Iran on 100 GBS isolated from pregnant and non-pregnant women who reported *rib *(53%) as the most prevalent alpha gene followed by *epsilon *(38%), *alp2/3 *(6%), and *alpha-c *(3%) (16). Another study in China on 193 GBS (invasive and noninvasive isolates) during a 7 year study showed the *rib* gene as the most prevalent *alp* gene at a rate of (45.1%), followed by *bca *(24.4%), *epsilon *(17.1%), *alp2/3* (3.6%), and *alp4* (1.0%), respectively (22). Various prevalence of *rib* encoding gene was reported regionally in Iceland (13.8%) (30), Iran (42.1%) (17), and China (8%) (5). These proteins, expressed by most if not all GBS strains contribute to GBS-related infections. Variability in the transmission of these genes in different studies is generally related to the possibility of transmission by horizontal transfer, changes in the expression of surface proteins that strongly correlate with clonal lineages, and the distribution of highly virulent clones in different geographic areas. All GBS isolates that contained Alp genes are thought to play a key role in the pathogenesis of GBS strains and are therefore potential vaccine candidates. Our data showed a high prevalence of PI -2a (75.7%), followed by PI -1 (70%) and PI -2b (24.3%) among GBS strains, which is not consistent with previous studies that identified PI -2a as the major pilus island gene in GBS strains (8). Our result regarding the prevalence of pilus island genes was similar to the results of previous studies in Ireland (31) and China (23). This can be explained by the fact that the occurrence of different molecular types of GBS in different areas is due to novel combinations of surface proteins, virulence factors, and pilus proteins.

The literature indicates that the distribution pattern of GBS-STs depends on several factors, including the geographic area, the population studied, and the source of GBS. In the present study, the 70 isolates belonged to 12 STs and were largely classified into the 3 main CCs reported worldwide. In a previous study by Jones and coworkers on 152 GBS strains isolated from seven countries, 29 STs were identified, of which about two-thirds belonged to 4 main STs, including ST -1, ST -17, ST -19, and ST -23. They showed a higher frequency of ST23 in both disseminated and invasive strains and also found a significant relationship between ST1, ST19, and asymptomatic dissemination and ST -17 and serotype III clones (32). Curiously, this work noted the occurrence of CC22 (40%), a very prominent GBS clone, in UTIs. In contrast, Bjoernsdóttir *et al*. reported a low prevalence rate of CC22 among their isolates (0.6%) in a study in Iceland (30). A previous study conducted by Pang and colleagues in China also showed the presence of CC22 isolates at a low level. The predominant prevalence of CC22 in the present study suggests that CC22 is the major genotype causing UTI associated with adult GBS and may be evidence of the emergence and rapid spread of this clone, likely due to importation from neighboring countries.

CC17 is one of the most common CC reported from different geographical areas. In the current work, a high prevalence of CC17 (25.7%) was found as the second most common genotype in GBS with a high prevalence of erythromycin resistance, confirming the previous findings of researchers in Kenya, who reported CC17 as the second most common CC (21.3%) in GBS strains isolated from hospitalized patients (33). This is supported by the observations of Kao *et al*. from Taiwan, who identified 11 different STs among the 182 isolates, most of which (56.6%) were ST17 (25), of which 89.3% were confirmed as erythromycin-resistant isolates. A previous report from China also identified the emergence of CC17 as the major genotype of the GBS strain causing invasive infections in neonates (34). They also reported that this CC as a hypervirulent genotype of GBS is the main type associated with pneumonia, purulent meningitis, and septicemia. Similarly, researchers in China reported CC17 as one of the detected CC in their study (23). 

In another research in Sweden, 158 GBS isolates associated with neonatal and adult invasive disease were investigated. Five STs 19, ST-17, ST-1, ST-23, and ST-9 were identified. A study reported the high frequency of CC19 and CC17 among isolates from adult and neonatal diseases, while CC17 significantly appeared to be associated with neonatal invasive disease (35). Alarmingly, the emergence of this CC plus various virulence encoding genes and simultaneously resistance to different antibiotics perhaps will be the trigger of its dissemination in Iran in the future.

Our results highlighted a relatively high frequency of CC23 (20%), as the third predominant type identified in GBS strains of UTI. The presence of this CC was reported in earlier studies conducted in Switzerland, Sweden, the UK, the USA, Italy, Taiwan, China, and Senegal (9, 12, 32). Results of drug resistance and virulence profile for CC23 isolate also displayed a high prevalence rate of CCR phenotype, resistance to tetracycline, and the majority of PI-1+PI-2a profile which contradicts the survey conducted by Björnsdóttir and coworkers in Iceland from 1975 to 2014, which reported a high frequency of CC23 with PI-2a and M phenotype (30). Multiple virulence and resistance profiles within CC23 clones were formerly reported by several investigators (30, 32). Our observations raise the possibility that CC23 isolates, although described earlier and in some of the geographic areas, have the potential to spread as a remarkable agent of UTI in Iranian adults (7, 10, 16). However, published data highlighted a significant relationship between ST17 and ST19 strains with the invasive disease while ST23 strains are related to asymptomatic colonization (5). 

Regardless of genetic variability, our work found a close correlation between virulence and resistance patterns and specific CC. All strains of CCs 22, 23, and nearly 90% of CC17 were tetracycline resistant and carried *tet*(M), *tet*(O), *tet*(K), *tet*(L), and *tet*(S) genes. Similarly, research demonstrated tetracycline resistance in the major GBS CCs, reflecting the important role of these tetracycline-resistant CCs in the development and spread of GBS disease (34). Previously, simultaneous transmission of virulence and resistance determinants was thought to reflect the selection of the best evolutionary lineages. The current study seems to confirm this theory. However, we did not find a strong relationship between virulence profile and specific STs or CCs, suggesting genetic heterogeneity in our clones.

Evidence suggests that there is a strong link between pilus island genes and the invasiveness of GBS strains. Meanwhile, there is evidence that these genes also have high immunogenicity. Therefore, expanding knowledge and awareness of PIs from different fields may be informative for future efforts to develop vaccines targeting pilus-derived proteins. We also demonstrated the presence of at least one PI in all GBS isolates, which is consistent with the results of previous studies (36, 37). Our data show that isolates in which PI -1+ PI -2a co-occur are mainly dependent on CC22, CC2323, and the ST74 and ST890 singletons. In our study, half of the CC17 isolates also carried PI -1 + PI -2b simultaneously. A similar result was found in the recent study by Khodaei *et al*. (2018), which showed that the majority of serotype III isolates carried PI -1 and PI -2a simultaneously (36). In agreement with our study, previous data published in South Africa showed that coexpression of PI -1 and PI -2b was associated with serotype III (37). In agreement with Khodaei *et al*. from Iran, the presence of PI -2b was lower than other PIs and distributed between ST52 and ST38 strains (36).

Singletons identified were ST74, ST38, and ST890. All had the ICR phenotype. Notably, ST890 was also reported in research performed by Cheng and colleagues in China on 72 GBS strains collected from pregnant women (28). This emergence has been reported by Kao *et al.* from Taiwan at rates of 1.6% (24). We reported ST74 and ST38, which had never been reported before.

**Table 1:** Distribution of phenotypic and genotypic resistance among CCs and STs of GBS strains

CC	ST	Resistance genes (No;%)	Phenotypic resistance (No;%)	Total N (%)
CC22	ST22	*erm*(B) (10; 52.6), *erm*(A) (10; 52.6), *mef*(A/E) (9; 47.4), *tet*(M) (19; 100), *tet*(O) (2; 10.5), *tet*(K) (2; 10.5),	ERY, LEV, TET (5; 26.3)	19 (27.1)
			ERY, TET, CHL, CEF (3; 15.8)	
			ERY, TET, CHL (4; 21.1)	
			ERY, CHL, TET, LEV (5; 26.3)	
			ERY, CEF (2; 10.5)	
	ST40	*erm(B) *(4; 66.7),* erm*(A) (4; 66.7), *tet*(M) (2; 33.3), *tet*(O) (2; 33.3), *tet*(K) (2; 33.3)	ERY, LEV, TET (2; 33.3)	6 (8.6)
			ERY, TET, CHL (4; 66.7)	
	ST189	*erm*(A) (2; 100), *tet*(S) (2; 100), *tet*(O) (2; 100)	ERY, LEV, TET (2; 100)	2 (2.9)
	ST155	*mef*(A/E) (1; 100), *tet*(M) (1; 100)	ERY, LEV, TET (1; 100)	1 (1.4)
CC17	ST17	*erm*(B) (9; 100), *tet*(M) (9; 100)	ERY, CLI, TET (9; 100)	9 (12.8)
	ST31	*erm*(B) (4; 66.7), *tet*(M) (4; 66.7)	CHL (2; 33.3)	6 (8.6)
			ERY, CLI, TET (4; 66.7)	
	ST48	*erm*(B) (3; 100), *tet*(M) (3; 100)	ERY, CLI, TET (3; 100)	3 (4.3)
CC23	ST23	*erm*(B) (12; 100),* mef*(A/E)(3; 25), *tet*(M) (9; 75), *tet*(L) (3; ), *tet*(O) (2; 25), *tet*(K) (1; 8.3)	ERY, CLI, TET (12; 100)	12 (17.1)
	ST52	*-*	CHL (2; 100)	2 (2.9)
Single ton	ST74	*erm*(B) (2; 50),* erm*(A) (2; 50)	ERY, CHL (2; 50)	4 (5.7)
			ERY (2; 50)	
	ST38	*erm*(A) (2; 100), *tet*(M) (2; 100), *tet*(O) (2; 100)	ERY, TET, CHL (2; 100)	2 (2.9)
	ST890	*erm*(A) (4; 100),	ERY (2; 50)	4 (5.7)
			ERY, CHL (2; 50)	

CCs: Clonal complexes, STs: Sequence types, GBS: Group B *Streptococcus*

STs: Sequence types, GBS: Group B *Streptococcus*, UTIs: Urinary tract infections

CCs: Clonal complexes, GBS: Group B *Streptococcus*, UTIs: Urinary tract infections

## Conclusion

Our main findings are i) the first report of ST74 and ST38 of GBS in Tehran, the capital of Iran, ii) the genetic diversity of GBS strains isolated from UTI with the majority of CC22, iii) the emergence of CC22 as the clone associated with ICR phenotype in Tehran, iv) the detection of CCR phenotype in the major types of CC17 and CC23. This study demonstrates the genetic variability of GBS isolated from urinary tract infections, with the majority of CC22 reflecting different clones circulating in Tehran, Iran. In addition, GBS isolated from adult UTIs were found to have a predominance of PI-1+PI -2a, *cylE*, *lmb*, *bca*, *rib*, and high resistance to tetracycline, erythromycin, and clindamycin, highlighting the need for surveillance and ongoing control of these strains. Given the high frequency of MDR patterns and the close relationship to specific clones in the GBS strains we studied, it is also strongly recommended to collect comprehensive data on molecular epidemiological patterns and antibiotic treatment according to drug resistance surveillance.

## Authors’ Contributins

M G and M R conceived and designed the study; M G, M R, M F, and CH A performed experiments and collected data; M G and M F analyzed and Interrelated results; M G supervised, directed, and managed the study; M G, M R, CH A, and M F approved the final version to be published.

## Conflicts of Interest

All authors declare that they have no competing interests. 
